# Gender Difference in Unmet Needs Among People Aging With a Traumatic Brain Injury

**DOI:** 10.1093/geroni/igab046.2665

**Published:** 2021-12-17

**Authors:** Kelli Barton, Emma Swinford

**Affiliations:** University of Missouri-Kansas City Institute for Human Development, Kansas City, Missouri, United States

## Abstract

Traumatic Brain Injury (TBI) can result in a myriad of short and long-term mental and physical changes and conditions. While fall-related brain injury prevention strategies and outcomes among older adults have been well-documented in previous literature, less is understood about the experiences and needs of those aging with a brain injury. The aim of this project is to explore gender differences in experiences and needs among people aging with a TBI. A Needs Assessment survey was conducted in early 2020 with adult TBI survivors and their family members in Missouri (n = 150). The mean age of respondents was 45.8 and 58% identified as male. Bivariate analyses reveal gender difference in unmet needs related to information and referral, recreation, and continuing education among TBI survivors. For example, more female respondents (43.1%) identified unmet needs associated with physical activity than their male counterparts (25.9%, p < .05). More females (61.3%) than males (43.4%) also identified unmet continuing education needs related to aging with brain injury (p < .05), whereas more males (10.8%) identified unmet continuing education needs on the topic of parenting (females: 1.6%, p < .05). Significantly more females (31.1%) than males (16.9%) identified lack of transportation as a barrier to accessing needed supports and resources (p < .05). Results will guide development of an Annual State Action Plan to maximize the independence, well-being, and health of Missourians aging with TBI and their families. A better understanding of needs and preferences can inform targeted policies, programs, and resources.

